# Evolution of the Autism Literature and the Influence of Parents: A Scientific Mapping in Web of Science

**DOI:** 10.3390/brainsci11010074

**Published:** 2021-01-08

**Authors:** Noemí Carmona-Serrano, Antonio-José Moreno-Guerrero, José-Antonio Marín-Marín, Jesús López-Belmonte

**Affiliations:** 1 Ceuta Autism Association, University of Granada, 51001 Ceuta, Spain; nhoe@correo.ugr.es; 2 Department of Didactics and School Organization, University of Granada, 51001 Ceuta, Spain; jesuslopez@ugr.es; 3 Department of Didactics and School Organization, University of Granada, 18071 Granada, Spain; jmarin@ugr.es

**Keywords:** autism, parent-based intervention, bibliometric analysis, scientific mapping, scimat, web of science

## Abstract

Parents interventions are relevant to address autism spectrum disorder (ASD). The objective of this study is to analyze the importance and evolution of ASD and its relationship with the parents (ASD-PAR) in the publications indexed in Web of Science. For this, a bibliometric methodology has been used, based on a scientific mapping of the reported documents. We have worked with an analysis unit of 1381 documents. The results show that the beginnings of scientific production date back to 1971. There are two clearly differentiated moments in scientific production. A first moment (1971–2004), where the production volume is low. A second moment (2005–2019), where the volume of production increases considerably. Therefore, it can be said that the subject began to be relevant for the scientific community from 2005 to the present. The keyword match rate between set periods marks a high level of match between periods. It is concluded that the main focus of the research on ASD-PAR is on the stress that is generated in families with children with ASD, in addition to the family problems that the fact that these children also have behavior problems can cause.

## 1. Introduction

Autism spectrum disorder (ASD) is conceived as a series of neurodevelopmental disorders with a multifactorial nature that affects 1.5% of the world population [1]. This disorder has alterations in the social plane [2], in the communicative aspects and in turn presents repetitive and stereotyped behaviors [3]. However, ASD is not limited only to that, but also these people have other deficiencies such as limitations in executive functioning, sensory perception and attention. They may also present depression, aggressiveness, challenging actions, emotional problems [4,5,6,7]. In this symptomatological line, there are also people with high levels of anxiety, which is aggravated if the person has a low cognitive level [8,9].

All of the above can be combined with another problem, such as an intellectual disability and an altered sensory system [10]. In this regard, the sense of touch stands out as a relevant element in human relationships, the condition of which causes disorders in the social aspect [11,12]. At a sensory level, people with ASD also present alterations in the reception of the sound around them [13], as well as in processing the visual stimuli of the environment [14]. However, the limitations of these people are not only here, on the sensory level. Moreover, both at the motor level [15], the use of language [16], the use of writing [17], and at the planning and structuring level of the tasks and actions of daily life [18], present limitations and alterations. However, not all of its capabilities are affected. People with ASD have greater precision in color processing [19] and greater processing of music than other typically developing people [20].

Expert literature reveals that the gender of these people can be an influencing factor in their conditions. In this sense, men reflect more restricted and repetitive behaviors and actions than women. This may be due to a disorder in brain structures, more specifically those related to social integration and cortico-striatum [21]. Despite these advances, there is currently no drug that contributes to improving all the disorders presented. Therefore, the intervention is postulated from a therapeutic perspective [22].

These interventions, if carried out early, can cause an important and significant improvement in the deficiencies of these people [23]. In addition, early intervention can enhance other types of unaffected skills [24,25]. This will contribute to a greater adaptation to the environment and to carrying out activities of daily life [26]. The nature of these interventions must be based on observation [27]. In all this, the family plays a fundamental role, as the agent or group of people closest to the person with ASD. Interventions carried out by the family can have a positive effect on these types of people [28]. Therefore, families value the fact of being involved in the therapeutic intervention process of their children [29]. This is currently being a focus under study [30]. However, family members’ knowledge of effective intervention guidelines is limited compared to other experts in this field of knowledge [31]. This lack of training can trigger behavioral patterns of social isolation [32]. Likewise, families can not only focus their daily activity on caring for the member with ASD, but they also have to provide financial support, so the workload is considerable [33]. Along these lines, families that have children with ASD experience higher rates of negativity than any other family [34].

Family training is positioned as a primary factor to achieve direct intervention to improve various indicators related to stress, depression, behavior problems, as well as improve the mental health of all members of the family unit [25]. These interventions must pursue quality rather than quantity [35]. State-of-the-art research postulates that adequate family cohesion can be beneficial to improving the quality of life of family members of people with ASD [36]. Likewise, the literature also reveals how companion animals can cause good results in reducing stress, both in people with ASD and in other members of the family unit [37].

### Justification and Objectives

This research analyzes the concept of “autism” in the parental environment (ASD-PAR) from a bibliometric perspective of the literature [38].

The Web of Science (WoS) has been taken as the database under study, as it is one of the largest databases in the world on social sciences. The novelty that this work assumes is the realization of an analysis of the documents published under an innovative technique of documentary study. In particular, in this research, a performance analysis and scientific mapping of the reported documents linked to the aforementioned terms has been carried out. In order to carry out bias-free research, the analytical structure of previous impact publications has been used to follow a study model validated by experts [39,40].

Specifically, this study is based on analyzing the significance and evolution of ASD-PAR in the publications indexed in WoS. It was started from an initial search in said database and no study was reported to the one presented in this work. Therefore, this research is raised under an exploratory nature in order to reveal to the scientific community and readers interested in the subject all the progress, evolution and upcoming trends [41]. This work will contribute to the reduction in the literary gap concerning the analyzed terms and will establish new knowledge bases on the state of the question, as well as start the path towards future works.

The objectives pursued by this study are: (a) to know the performance of the scientific production on ASD-PAR in WoS; (b) to determine the scientific evolution on ASD-PAR in WoS; (c) to discover the most relevant topics about ASD-PAR in WoS; (d) to locate the most representative authors on ASD-PAR in WoS.

## 2. Materials and Methods

### 2.1. Research Design

For the development of the study and the subsequent achievement of the objectives, a bibliometric research design was used. This methodology bases its potential in quantifying and comprehensively evaluating scientific documentation [42,43]. The design presented in this work will allow the efficient search, registration, analysis and prediction of the existing literature on the subject [44].

Specifically, the design has been based on a co-word analysis [45], as well as the study of various indices (h, g, hg and q2) [46]. The h index is an indicator that is used to measure the quality of the scientists’ production according to the number of citations received in their publications. The g index allows us to delve into the productive analytics of researchers who have a similar value in the h index. The hg index is a combination of the previous indices. It allows us to obtain a result that takes into account the potentialities of the indices “h” and “g” and reduces their drawbacks. Finally, the q2 index is prepared from a quantitative measure (h index) and another based on the qualitative properties of the h nucleus [47,48].

The investigative actions carried out will allow the generation of maps with nodes to represent the performance, the location of the subdomains of the concepts and the development of the linked topics [49] on ASD-PAR in the WoS database.

### 2.2. Procedure

The research has been carried out in various phases following the considerations and protocols of the specialists to carry out a pertinent and methodical study [50,51,52,53]. The first action was to select the database (WoS). Then, the search concepts were specified (autism, parents, father and mother). Next, the search equation was created: “autism” (TITLE) AND “mother *” OR “father *” OR “parents” (TITLE). Next, the search process was carried out in the main WoS collection, in the indices SCI-EXPANDED, SSCI, A & HCI, CPCI-S, CPCI-SSH, BKCI-S, BKCI-SSH, ESCI, CCR-EXPANDED and IC. The starting date of the search is 1900, which is the time when the database starts to collect manuscripts.

**Figure 1:**
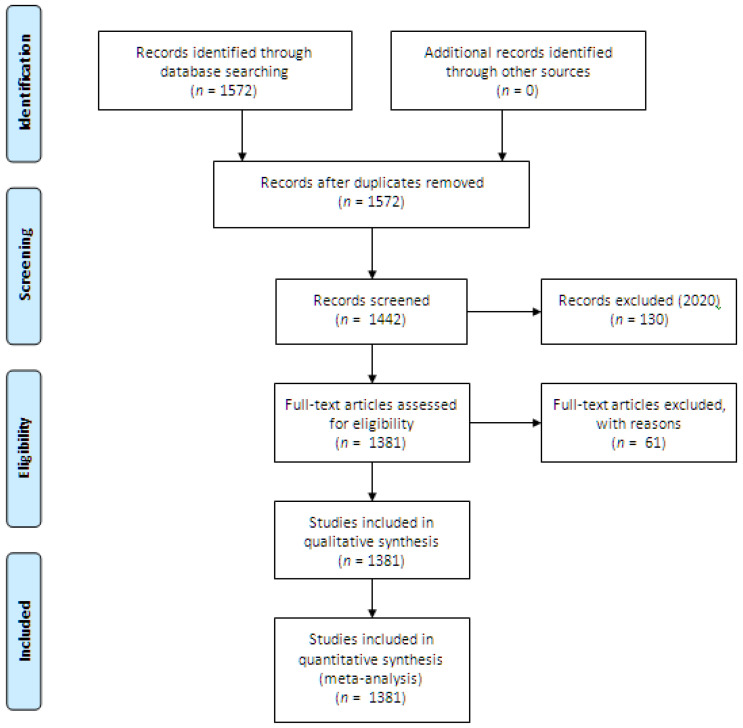
Flowchart according to the PRISMA Declaration.

Once the actions of each phase had been carried out, a total of 1572 publications were reported. This initial number of documents was refined through the establishment of various criteria [54,55]. The exclusion criteria were: 1—Documents published in 2020. This is because the year has not yet ended and new documents dated 2020 may be included in the coming months (*n* = 130); 2—Repeated or poorly indexed documents in WoS (*n* = 61). After applying these criteria, the final unit of analysis was established in 1381 publications. Figure 1 synthesizes in a flow diagram the actions deployed following the PRISMA protocol.

In order to present the results of scientific performance and production, a series of inclusion criteria have been established, which were delimited in: 1—Year of publication (all production except 2020). The search began in 1900. The first manuscript on this subject appeared in 1971; 2—Language (*x* ≥ 20); 3—Publication area (*x* ≥ 100); 4—Type of documents (*x* ≥ 100); 5—Organizations (x ≥ 29); 6—Authors (*x* ≥ 15); 7—Sources of origin (*x* ≥ 40); 8—Countries (*x* ≥ 100); 9—The four most cited documents (*x* ≥ 350).

### 2.3. Data Analysis

The tools used to perform the data analysis were Analyze Results, Creation Citation Report (programs to collect the year, authorship, country, type of document, institution, language, medium and most cited documents) and SciMAT (program to carry out the structural and dynamic development of the documents reported from a longitudinal perspective). For a correct performance of the tools, the considerations of other previous works were followed [56,57].

With SciMAT, likewise, a co-word analysis was carried out that covered various processes [58]. In the recognition process, the keywords (*n* = 3080) of the entire document package of the unit of analysis were studied. Afterwards, the co-occurrence node maps were designed. Next, a normalized network of co-words was generated and the most significant keywords were selected (*n* = 2887). Moreover, the most relevant topics and concepts were compiled with a clustering algorithm. In the process of reproduction, different thematic networks and strategic diagrams articulated in four quadrants were created. Each quadrant, depending on its location, presents a different meaning (upper right = motor and relevant themes; upper left = deep-rooted and isolated themes; lower left = disappearing or projected themes; lower right = themes of little development and cross-cutting). The principles of density and centrality intervened in this process. Density measures the internal strength of the network. Centrality measures the level of connection of a network with others [59]. For the determination process, the literature reported in different periods was configured. All this to analyze the evolution of the nodes in different time intervals. In this work, three periods have been established (*P*_1_ = 1971–2012; *P*_2_ = 2013–2016; *P*_3_ = 2017–2019). These intervals have been established under the criterion of documentary similarity between the different periods. To determine the associative strength between the periods, the number of keywords they contained in common was used as a reference. On the other hand, for the analysis of authorship, only an interval was established that covers the entire time period that has marked the publication report (*P*_*X*_ = 1971–2019). Finally, in the performance process, various production indicators connected to their corresponding inclusion criteria were defined [60]. The analysis unit determines the unit of valuation on the keywords established by the authors of the publications, as well as the keywords established by WoS. The frequency threshold reflects the minimum frequency threshold for keywords that are repeated in each time interval. The network type refers to the network to be configured (co-occurrence network). The threshold of the co-occurrence union value establishes the marked periods, according to authors and keywords. The normalization measure determines the connection threshold, determining the minimum relationship for co-occurrence. The normalization measure reveals the measure to normalize the network. For this, the equivalence index *eij* was used. This is calculated as follows: *eij = cij2/Root (ci−cj)*. In a disaggregated manner, *cij* is the number of coincidences of *i* and *j* in the set of documents, *ci* is the number of occurrences of *i*, and *cj* is the number of occurrences of *j*. On the other hand, the clustering algorithm is used to elaborate the map and its links. The evolutionary measure determines the degree of similarity necessary to elaborate the evolution map, which is established with the Jaccard index. Finally, for the transition map, the inclusion rate is used. All these parameters have served for the optimal configuration of SciMAT (Table 1).

## 3. Results

### 3.1. Scientific Performance and Production

**Figure 2:**
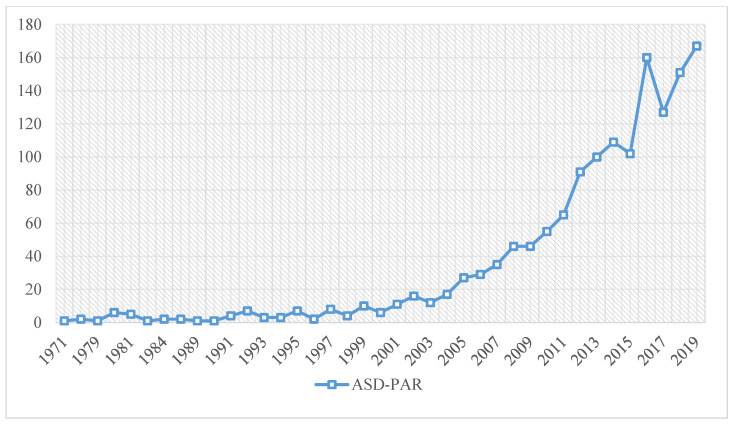
Evolution of scientific production. Note: *Y*-axis: number of manuscripts; *X*-axis: dates of publication.

The evolution of manuscript production in the scientific field of ASD-PAR has two clearly differentiated moments. Although the search began in 1900, it was not until 1971 that the first manuscripts appeared under the theme of this study. From that date until 2004, the volume of production is relatively low, not exceeding 20 documents per year. In the second period, which runs from 2005 to 2019, the number of scientific productions increases gradually and considerably until the present day. Only one evolutionary anomaly is observed between 2015 and 2017, where there are downward and upward peaks in scientific production (Figure 2).

The language used in manuscripts about ASD-PAR is mainly English. It is followed, by far, by French (Table 2).

The area of knowledge that houses research on ASD-PAR is developmental psychology, although it is closely followed by other areas of knowledge, such as psychiatry and rehabilitation (Table 3).

The type of document used to present the research results are research articles. This type of document is far from the other typologies (Table 4).

The main institution in this line of research is the University of California System, although it is closely followed by the Universities of Wisconsin (Table 5).

There are three authors who stand out in this line of research, namely Seltzer, M.M., Ekas, N.V. and Hastings, R.P., with regard to the volume of production (Table 6).

Of all the journals compiling studies on ASD-PAR, the Journal of Autism and Developmental Disorders stands out very considerably in terms of volume of production (Table 7).

The country with the highest production volume over ASD-PAR is the United States, being far away from the rest of the countries (Table 8).

The four most frequently cited manuscripts on ASD-PAR (Table 9) refer to parental stress in families with young children with ASD with an average age of 26.9 months [61], to the higher stress in families with students with ASD than other families with children with other symptoms [62], to the level of well-being in families with students with ASD, where it is higher in relation to disabilities such as Down’s or Fragile X syndrome [63], or that those families with students with ASD who present behavioral problems show higher levels of stress than those families with children with ASD who do not show behavioral problems [64].

### 3.2. Structural and Thematic Development

**Figure 3:**
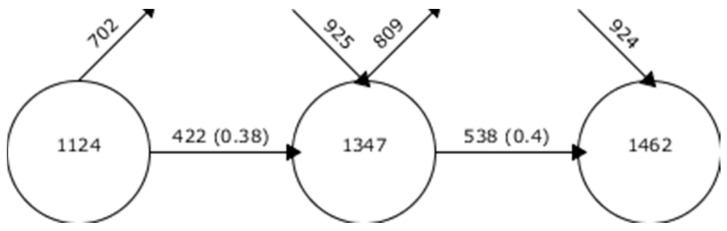
Continuity of keywords between contiguous intervals.

The evolution of keywords shows the development of research on a subject of study according to the keywords used by the authors. In this case, one can observe the keywords that have been used in a specific period, the keywords that are no longer used in a specific period, the new keywords that are used in a specific period and the keywords that coincide between contiguous periods. As can be seen in Figure 3, the percentage of coincidence between periods is high, being close to 40%. This indicates that research on ASD-PAR is based on similar lines of research, given that there is coincidence between researchers.

The study of the academic performance of a given field of study analyses the various bibliometric values shown by the research topics. In this case, in the first period (1971–2012) the subjects with the most bibliometric values are “Young-children” and “stress”. In the second period (2013–2016), the themes with the most bibliometric values are “behavior-problems” and “families”. In the third period, the theme with the most bibliometric values is “mothers” (Table 10).

**Figure 4:**
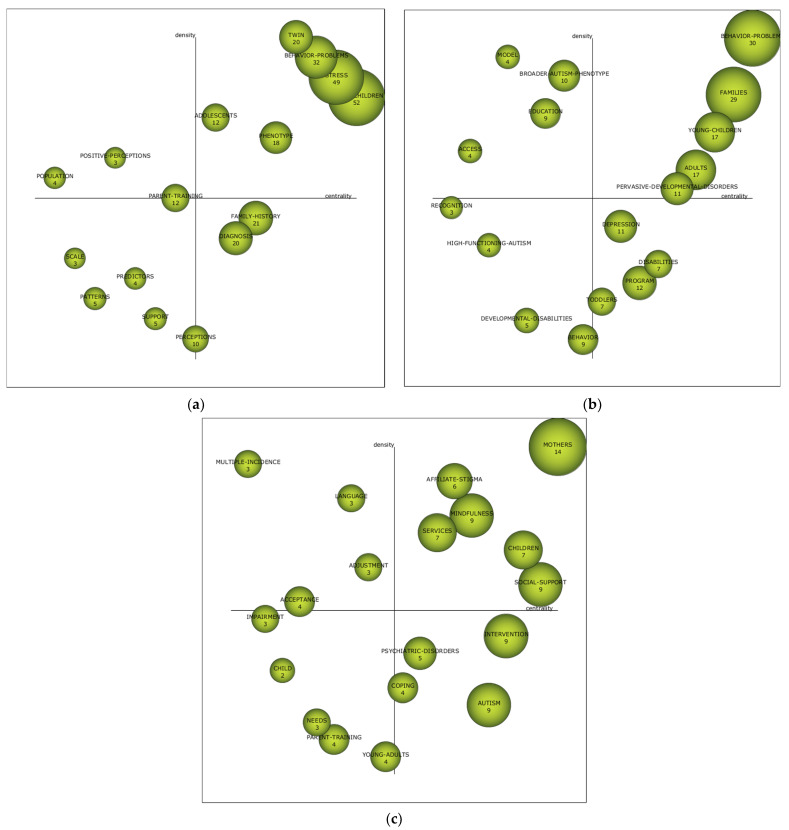
Strategic diagram per ASD-PAR index-h. Note: (**a**) Interval 1971–2012; (**b**) Interval 2013–2016; (**c**) Interval 2017–2019.

Strategic diagrams provide information on the relevance of a theme in a given time period. Figure 4 shows the position of the different themes, showing the index h, and taking into account both the external connection force (centrality) and the internal connection force (density).

In the first period (1971–2012), the driving themes are “adolescents”, which are related to “adults”, “depressed-Mood”, “expressed-emotion”, “quality”, “validity”, “reliability”, “schizophrenia” and “symptoms”; “phenotype, which relates to “brain”, “deficits”, “disorders”, “personality-characteristics”, “pervasive-developmental-disorders”, “psychiatric-disorders”, and “weak-central-coherence”; “twin”, which relates to “broad-autism-phenotype”, “children”, “genetics”, “history”, “individuals”“infantile-autism”, “personality” and “traits”; “behavior-problems”, which relates to “fathers”, “family-stress”, “intellectual-disability”, “maternal-stress”, “mental-health”, “parenting-stress”, “preschool-children” and “syndrome-specificity”; “stress”, which relates to “adjustment”, “coping”, “depression”, “families”, “health”, “mothers”, “parents” and “social-support”; and “Young-children”, which relates to “autism”, “behavior”, “communication”, “disabilities”, “Down-Syndrome”, “intervention”, “mental-retartion” and “spectrum-disorders”. In this period, research is focused on the behavioral problems of children with ASD, stress in families, in young children and adolescents.

In the second period (2013–2016), the motor themes are “behavior-problems”, which is related to “Down-Syndrome”, “intellectual-disability”, “mental-health”, “mothers”, “parenting-stress”, “preschool-children”, “stress” and “syndrome-specificity”; “families”, which is related to “adjustment”, “autism”, “autism-spectrum-disorders”, “fathers”, “impact”, “marital-satisfaction” and “parents”; “Young-children”, which relates to “double-abcx-model”, “intervention”, “joint-attention”, “meta-analysis”, “school-age-children”, “skills”, “social-support” and “support”; “adults”, which relates to “adolescents”, “Asperger-syndrome”, “children”, “gender”, “health”, “positive-perceptions”, “prevalence” and “validity”; and “pervasive-developmental-disorders”, which relates to “coping-strategies”, “diagnosis”, “parental-stress”, “population”, “randomized-controlled-trial”, “spectrum-disorders”, “symptom-severity” and “traits”. During this period, the focus is on behavioral problems, families, and people of various ages with ASD and generalized developmental disorders.

In the third period (2017–2019), the motor themes are “mothers”, which is related to “autism-spectrum-disorder”, “families”, “fathers”, “mental-health”, “parents”, “preschool-children”, “quality-of-life” and “stress”; “affiliate-stigma”, which relates to “stigma”, “people”, “caregivers”, “family-caregivers”, “intellectual-disability”, “perceptions” and “psychological-distress”; “services”, which relates to “advocacy”, “anxiety”, “awareness”, “behaviour-problems”, “care”, “decreases-aggression”, “depression”, “disparities”, “education”, “health”, “health-care” and “meta-synthesis”; “mindfulness”, which relates to “parent-intervention”, “program”, “stress-reduction” and “therapy”; “children”, which relates to “adolescents”, “ASD”, “diagnosis”, “individual”, “prevalence”, “risk-factors”, “transition” and “youth”; and “social-support”, which relates to “ASD”, “developmental-disabilities”, “disabilities”, “Down-Syndrome”, “parenting-stress”, “impact”, “predictors” and “satisfaction”. In other words, during this period, the focus is more on the care, services and social support that families and people with ASD can receive. In addition, the themes of “child”, “needs”, “parent-training” and “Young-adults” must be taken into account during this period, as they are considered to be unknown themes. In other words, they may disappear from the lines of research, or become the driving forces of the coming years in the field of research.

### 3.3. Thematic Evolution of Terms

The thematic evolution of a field of knowledge shows the relationship that is established between the different subjects in contiguous periods. This gives an idea of the different lines of research established in a specific research topic. The type of relationship that can be established between the topics can be conceptual and non-conceptual. The conceptual relationship occurs when two themes share common constructs. The non-conceptual relationship is generated when the two topics do not share keywords in common. The conceptual relationship is represented by a solid line. The non-conceptual relationship is shown with dashed lines. The size of the line indicates the number of relationships (the thicker the line, the greater the relationship).

**Figure 5:**
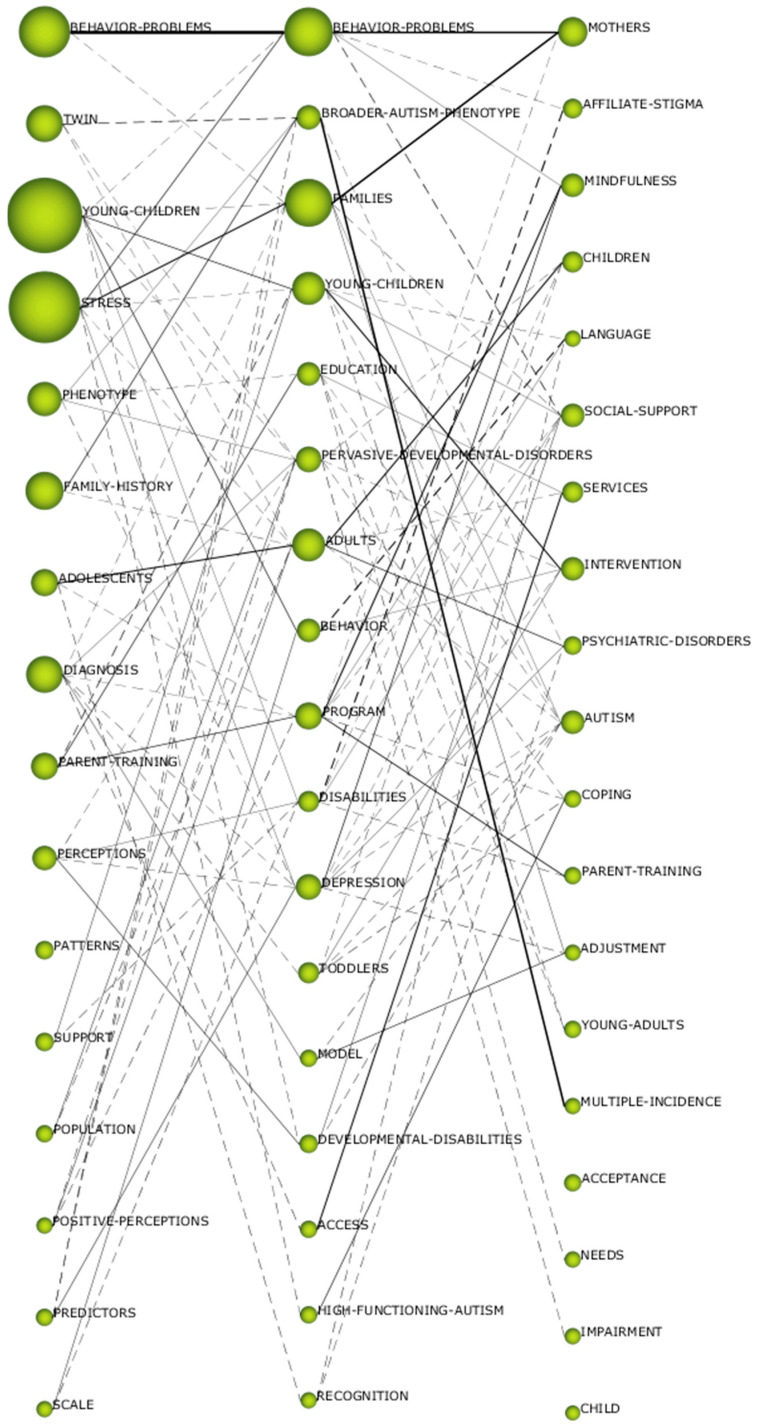
Thematic evolution by h-index.

In the field of study of ASD-PAR, it can be indicated that a conceptual gap exists. In other words, there is not one theme that is repeated in all three periods. This indicates a variety of themes in the fields of study undertaken. This does not mean that there are not diverse lines of research over time. In this case, two can be highlighted, on the one hand the line “behavior_problems-behavior_problems-mothers” and “stress-families-mothers”. That is, the lines of research established over time focus mainly on behavioral problems and their repercussions on families, and on the stress generated in the family environment by living with a person with ASD. In addition, Figure 5 shows that there are more conceptual than non-conceptual relationships, which shows the strong relationship between the various topics. It can also be seen that between the second and third periods several lines of research are being established, which may set the trend for study in the coming years.

### 3.4. Authors with the Highest Relevance Index

**Figure 6:**
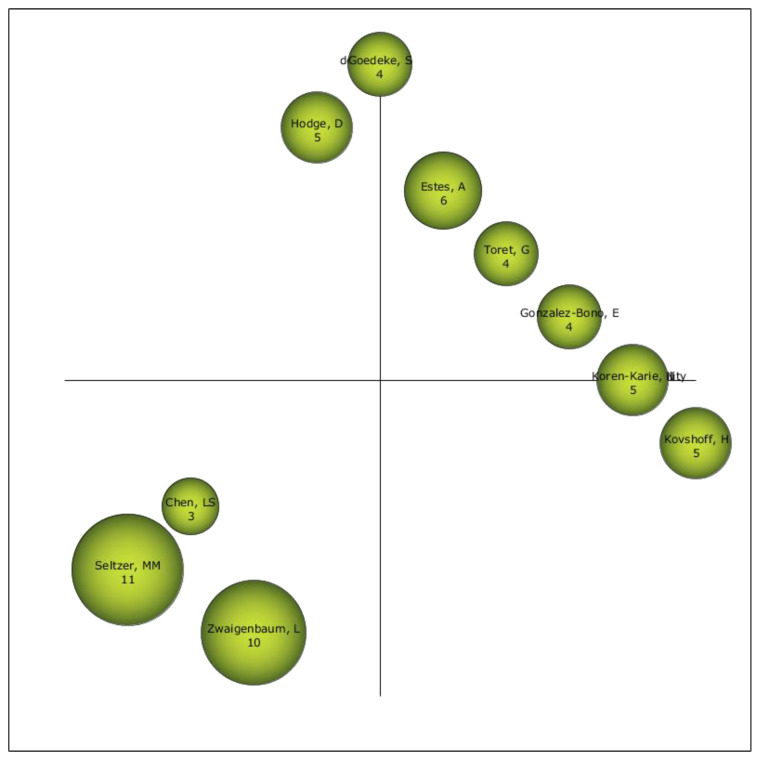
Strategic author diagram of the entire production.

Taking into account the authors in the ASD-PAR field of study, it can be indicated that those considered as drivers are Estes, A., Toret, G. and González-Bono, E. Although, due to their location in the diagram, we must take into consideration Chen, L.S., Seltzer, M.M. and Zwaigenbaum, L., because they may become the relevant authors in this field of study (Figure 6).

## 4. Discussion

The actions of family members acquire a relevant value when it comes to intervening and treating people with ASD [28,29]. The literature shows a pronounced interest in carrying out studies that represent an advance in this field of knowledge [30]. In this sense, research reports effective mechanisms and actions to address this disorder in the best possible way [25,31,32,33,34,35,36,37]. With the completion of this study, we have tried to analyze all the literature concerning ASD and the family environment, in order to report the most significant and relevant findings that the scientific community has obtained on the state of the matter.

The performance and production analysis on ASD-PAR in WoS allows to establish a profile on this field of study. In this case, it can be indicated that the beginnings of scientific production in Wos date back to 1971. There are two clearly differentiated moments in scientific production: a first moment (1971–2004), where the volume of production is low, and a second moment (2005–2019), where the volume of production increases considerably. It can therefore be said that the subject matter began to be relevant for the scientific community from 2005 to the present day. The manuscripts are presented in the form of articles, which indicates that this field of study is well established in the scientific community, in English and in the Journal of Autism and Developmental Disorders. The area of knowledge where this type of study is compiled is developmental psychology. Analyzing the following areas of knowledge, it can be seen that studies on ASD-PAR are oriented towards the psychological and rehabilitative support of families who have children or relatives with ASD. The main institution conducting research on ASD-PAR is the University of California System. However, the volume of the University of Wisconsin System is noteworthy in this regard. In this case, the first places are occupied by universities in the United States, which happens to be the country with the highest volume of production. In relation to the authors, it is necessary to bear in mind several premises. On the one hand, there are those with a higher rate of production, including Seltzer, M.M., Ekas, N.V. and Hastings, R.P. On the other hand, there are the most relevant within the scientific community, in this case Estes, A., Toret, G. and González-Bono, E. Finally, the authors Chen, L.S., Seltzer, M.M. and Zwaigenbaum, L., should be taken into consideration as they are probably the most relevant in the near future. The most cited article is by [56] and the lines of research of the most cited articles focus on the stress and well-being of families with children with ASD.

The index of key word coincidence between the established periods marks a high level of coincidence between periods, which shows a high degree of agreement on the existing lines of research on the subject of ASD-PAR. The academic performance indicates that there is no single subject that presents high bibliometric values in the three periods analyzed. In general, it is shown that stress, behavioral problems, families and mothers are the most relevant research topics in the field of study of ASD-PAR.

The study developed also indicates that there is no theme that is repeated, as a motor theme, in the three established time diagrams. However, similar themes of study are visualized, which focus on stress in the family, mothers, behavioral problems of people with ASD and the problems of people with ASD at different ages. This is specifically reflected in each of the established time periods. In the first period (1971–2012), motor issues were focused on “Twin”, “behavior-problems”, “stress”, “young-children”, “adolescents” and “phenotype”. In other words, on the behavioral problems presented by children with ASD, the stress of families and on the children and adolescents themselves. In the second period (2013–2016), the motor themes were oriented towards “behavior-problems”, “families”, “young-children”, “adults” and “pervasive-developmental-disorders”. This means behavioral problems, families, people of various ages with ASD and generalized developmental disorders. In the last period (2017–2019) the driving themes are “mothers”, “affiliate-stigma”, “mindfulness”, “services”, “children” and “social support”. In other words, in this period, the driving themes are more oriented towards the care, action services and social support that families and people with ASD can receive.

If we look at the thematic evolution of the studies on ASD-PAR, we can see that there is a conceptual gap, although two clearly defined and time-based lines of research can be observed, such as “behavior_problems-behavior_problems-mothers” and “stress-families-mothers”. In this case, it can be said that the research is oriented towards behavioral problems and their impact on families, and the stress generated in the family environment by living with a person with ASD.

## 5. Conclusions

It is concluded that the field of study on ASD-PAR began in WoS in 1971, but it was not until 2005 that it began to be relevant and interesting for the scientific community. The main focus of research on ASD-PAR is on the stress that is generated in families that have children with ASD, in addition to the family problems that can result from the fact that these children also have behavioral problems.

The limitations of this study focus on the purification of the database, since the researchers of this manuscript have had to read each of the documents, in order to properly apply the PRISMA protocol. Another of the limitations focuses on the debugging of the database, given that badly written or badly expressed key words must be modified or eliminated. Finally, the inclusion criteria can be considered, which have been established from the perspective of the researchers themselves, from their experience and with the intention of showing the most relevant information on this field of study. The future line of research derived from this investigation focuses on developing studies focused on families who have children with ASD, broadening the field of knowledge.

## 6. Theoretical and Practical Implications

This study has a number of theoretical and practical implications. Among the theoretical implications are the broadening of the field of knowledge about ASD-PAR. Until now, no such research has been carried out. This research provides clear information on the lines of study established by the scientific community. In addition, it presents a specific profile of this type of research. Another of the theoretical implications is that the most recent research has been compiled in order to prepare the introduction to this manuscript, offering up-to-date and high-impact information on the research carried out in this field of study. Amongst the practical implications, this work allows those groups responsible for attending to families with children with ASD to provide information on where the lines of study are heading, as well as offering data on the most relevant authors in this field of knowledge. Likewise, in the development of the different analyses, information is offered on various methods and actions to attend to families with children with ASD.

## Figures and Tables

**Table 1:** Production indicators and inclusion criteria.

Configuration	Values
Analysis unit	Keywords authors, keywords WoS
Frequency threshold	Keywords: *P*_1_ = (4), *P*_2_ = (4), *P*_3_ = (4)
	Authors: *P_X_* = (5)
Network type	Co-occurrence
Co-occurrence union value threshold	Keywords: *P*_1_ = (2), *P*_2_ = (2), *P*_3_ = (2)
	Authors: *P_X_* = (3)
Normalization measure	Equivalence index: *eij = cij2/Root (ci−cj)*
Clustering algorithm	Maximum size: 9; Minimum size: 3
Evolutionary measure	Jaccard index
Overlapping measure	Inclusion Rate

**Table 2:** Scientific language of publications.

Languages	*n*
English	1380
French	22

**Table 3:** Areas of knowledge.

Areas of Knowledge	*n*
Psychology developmental	496
Psychiatry	345
Rehabilitation	335
Education Special	289

**Table 4:** Type of document.

Type of Document	*n*
Article	1011
Meeting abstract	170
Book review	141

**Table 5:** Institutions.

Institutions	*n*
University of California System	53
University of Winconsin System	44
University of North Carolina	30

**Table 6:** Most prolific authors.

Authors	*n*
Seltzer, M.M.	17
Ekas, N.V.	16
Hastings, R.P.	16

**Table 7:** Source of origin.

Source	*n*
Journal of Autism and Developmental Disorders	157
Autism	89
Research in Autism Spectrum Disorders	63
Journal of Intellectual Disability Research	47

**Table 8:** Most productive countries.

Countries	*n*
USA	606
England	143
Australia	127
Canada	111

**Table 9:** Most cited articles on autism spectrum disorder and its relationship with parents (ASD-PAR).

References	Citations
[61]	505
[62]	448
[63]	420
[64]	350

**Table 10:** Thematic performance in ASD-PAR.

**Interval 1971–2012**						
**Denomination**	**Works**	**Index h**	**Index g**	**Index hg**	**Index q2**	**Citations**
Adolescents	15	12	15	13.42	23.75	582
Behavior-problems *	44	32	44	37.52	51.54	4423
Diagnosis	24	20	24	21.91	31.3	1145
Family-history	23	21	23	21.98	37.79	1966
Parent-training *	17	12	17	14.28	22.45	778
Patterns	5	5	5	5	13.96	341
Perceptions	11	10	11	10.49	18.44	521
Phenotype	22	18	22	19.9	30.59	1196
Population	4	4	4	4	14.83	209
Positive-perception	3	3	3	3	12.96	227
Predictors	4	4	4	4	25.69	547
Scale	3	3	3	3	6	32
Stress	106	49	84	54.16	69.3	7316
Support	6	5	5	5	20	385
Twin	25	20	25	22.36	41.95	1914
Young-children *	107	52	89	68.03	69.17	8102
**Interval 2013–2016**						
**Denomination**	**Works**	**Index h**	**Index g**	**Index hg**	**Index q2**	**Citations**
Access	5	4	5	4.47	13.71	140
Adults	44	17	28	21.82	22.2	878
Behavior	17	9	16	12	13.42	312
Behavior-problems *	127	30	52	39.5	40.62	3360
Broader-autism-phenotype	14	10	14	11.83	14.83	254
Depression	16	11	16	13.27	17.55	498
Developmental-disbilities	6	5	6	5.48	8.06	191
Disabilities	16	7	14	9.9	10.58	257
Education	13	9	13	10.82	11.62	198
Families	150	29	51	38.46	39.2	3369
High-functioning-autism	5	4	4	4	10.77	125
Model	5	4	5	4.47	9.38	71
Pervasive-develpmental-disorders	20	11	18	14.07	16.58	556
Program	19	12	19	15.1	18	460
Recognition	3	3	3	3	11.75	144
Toodlers	9	7	9	7.94	10.25	191
Young-children *	43	17	35	24.39	23.32	1309
**Interval 2017–2019**						
**Denomination**	**Works**	**Index h**	**Index g**	**Index hg**	**Index q2**	**Citations**
Acceptance	5	4	5	4.47	6.63	44
Adjustment	8	3	6	4.24	5.74	59
Affiliate-stigma	20	6	10	7.75	8.12	122
Autism	47	9	13	10.82	12	276
Child	3	2	3	2.45	3.46	12
Children	70	7	11	8.77	10.58	267
Coping	9	4	5	4.47	5.66	36
Impairment	3	3	3	3	3	15
Intervention	66	9	12	10.39	10.39	300
Language	13	3	5	3.87	4.58	35
Mindfulness	30	9	13	10.82	10.82	229
Mothers	220	14	19	16.31	17.15	969
Multiple-incidence	6	3	4	3.46	7.75	49
Needs	4	3	3	3	3.46	13
Parent-training *	9	4	7	5.29	7.21	57
Psychiatric-disorders	16	5	8	6.32	5.92	76
Services	20	7	10	8.37	9.17	122
Social-support	72	9	13	10.82	10.39	363
Young-adults	7	4	7	5.29	8.94	70

Note: (*): Themes repeated in different periods.

## Data Availability

Data is contained within the article.
